# Supplementary material for: Update on frequency decline of Northeast Pacific blue whale (Balaenoptera musculus) calls
Source: PLoS One. 2022 Apr 1;17(4):e0266469. doi: 10.1371/journal.pone.0266469 (PMC8975115; doi:10.1371/journal.pone.0266469)
Supplement: S1 Table — The year, site, latitude, longitude, depth, and analysis period for each recording package deployment are provided, along with the associated mean frequency (and pulse rate for A calls) and 95% confidence intervals (CI) for both A and B calls each year. (PDF) [file pone.0266469.s001.pdf]

S1 Table. High-frequency acoustic recording package deployment details.

Year	Site	Latitude (N)	Longitude (W)	Depth (m)	Analysis period	A call				B call	
						Mean frequency (Hz)	95% CI	Mean pulse rate (pulses s^{-1})	95% CI	Mean frequency (Hz)	95% CI
2006	E	32° 39.1’	119° 28.9’	1330	09/22/06–10/31/06	85.6	0.43	1.241	0.016	46.1	0.15
2007	E	32° 39.4’	119° 28.4’	1316	09/11/07–10/31/07[*]	85.6	0.36	1.219	0.023	46.0	0.13
2008	H	32° 50.8’	119° 10.5’	1017	09/05/08–10/31/08	84.7	0.46	1.221	0.013	45.5	0.12
2009	N	32° 21.2’	118° 33.9’	1295	09/28/09–10/31/09	84.9	0.55	1.181	0.031	45.3	0.10
2010	N	32° 22.2’	118° 33.8’	1288	09/28/10–10/31/10	84.3	0.74	1.185	0.020	44.9	0.12
2011	H	32° 50.6’	119° 10.3’	1000	09/18/11–10/31/11	84.1	0.48	1.197	0.012	44.6	0.13
2012	N	32° 22.2’	118° 33.9’	1285	09/21/12–10/31/12	83.4	0.31	1.177	0.015	44.2	0.16
2013	H	32° 50.3’	119° 10.0’	1000	09/22/13–10/31/13	83.0	0.39	1.187	0.010	44.0	0.16
2014	N	32° 22.2’	118° 33.8’	1260	09/22/14–10/31/14	82.6	0.46	1.168	0.010	43.6	0.16
2015	N	32° 22.2’	118° 33.8’	1260	09/25/15–10/31/15	82.0	0.44	1.161	0.015	43.4	0.13
2016	N	32° 22.3’	118° 33.9’	1200	09/22/16–10/31/16	81.6	0.50	1.176	0.019	43.3	0.13
2017	E	32° 39.5’	119° 28.8’	1312	09/27/17–10/31/17	82.0	0.40	1.176	0.010	42.9	0.14
2018	E	32° 39.5’	119° 28.6’	1300	09/22/18–10/31/18	81.4	0.43	1.151	0.016	42.9	0.14
2019	H	32° 51.7’	119° 08.4’	1270	09/01/19–10/31/19	81.5	0.45	1.158	0.009	42.6	0.12

The year, site, latitude, longitude, depth, and analysis period for each recording package deployment are provided, along with the associated mean frequency (and pulse rate for A calls) and 95% confidence intervals (CI) for both A and B calls each year.

[*]Deployment with a 5-min on and 15-min off duty cycle.
