# Supplementary material for: Update on frequency decline of Northeast Pacific blue whale (Balaenoptera musculus) calls
Source: PLoS One. 2022 Apr 1;17(4):e0266469. doi: 10.1371/journal.pone.0266469 (PMC8975115; doi:10.1371/journal.pone.0266469)
Supplement: Attachment — Submitted filename: Review.pdf [file pone.0266469.s004.pdf]

Notes on “Update on Frequency decline of NE Pacific blue whale calls”

This paper measures the peak frequency of type A and B calls collected on the Southern California Bight between 2006 and 2019 and reproduces an earlier reported result that the frequency of type B calls is decreasing gradually over time, albeit at a slower rate than reported for earlier times. The paper also measures the frequency shift of the A call, which is a tricker measurement due to the pulsive nature of the call.

The content of the paper is interesting and should be eventually published, but the statistical regression analysis is too simplistic and should be expanded to control for other factors that might affect the received peak frequency. Furthermore, while the authors provide data on the pulse rate of type A calls (Table S2), the paper performs no analysis on this pulse rate, which is unfortunate, as the pulse rate of the calls also seems to be decreasing over time, as the attached plot shows. The relatively lean results of this manuscript would be enhanced if pulse rate were also included in the analysis.

(1) Statistical regression: The paper performs a simple least-squares fit to the frequency data of both types of calls. Unfortunately, apparent peak frequency can be affected by factors other than the source (such as range and instrumentation), and the dataset is large enough that some of these factors can be checked. At a minimum the authors should use a generalized linear model (GLM) using received level as a predictor variable, HARP unit/location as a categorical predictor variable, and year as another predictor variable. The importance of received level is that it is a proxy for horizontal range, and there is abundant evidence that range and environmental propagation conditions impact peak frequency in other animals. Even if the water depths here seem deep (1 km in some locations), frequency-dependent attenuation and bottom interactions cannot be ruled out.

In addition, the paper should clarify whether the FFT length used to analyze the peak frequency of the type A call was shorter than the pulse rate. If the FFT length is too long, then the spectrum of the type A call will shift from the structure shown in Figure 2 to a “comb-like” spectrum where the separation between comb peaks is the reciprocal of the pulse rate. It’s important to ensure that the 75 Hz frequency peak measured is due to the “formant” shift and not from changes in the pulse rate.

(2) Pulse rate analysis: the attached figure, generated from supplemental table S2, shows that the pulse rate of type A calls is decreasing over time, to the point that by 2019 the rate has shifted one standard deviation from 2006. This would suggest that a more rigorous regression would find a significant relationship between pulse rate and time, an observation that may have a bearing on the extensive discussion.

(3) About that extensive discussion: I would make it shorter, as a detailed list of speculations does not enhance the paper quality.

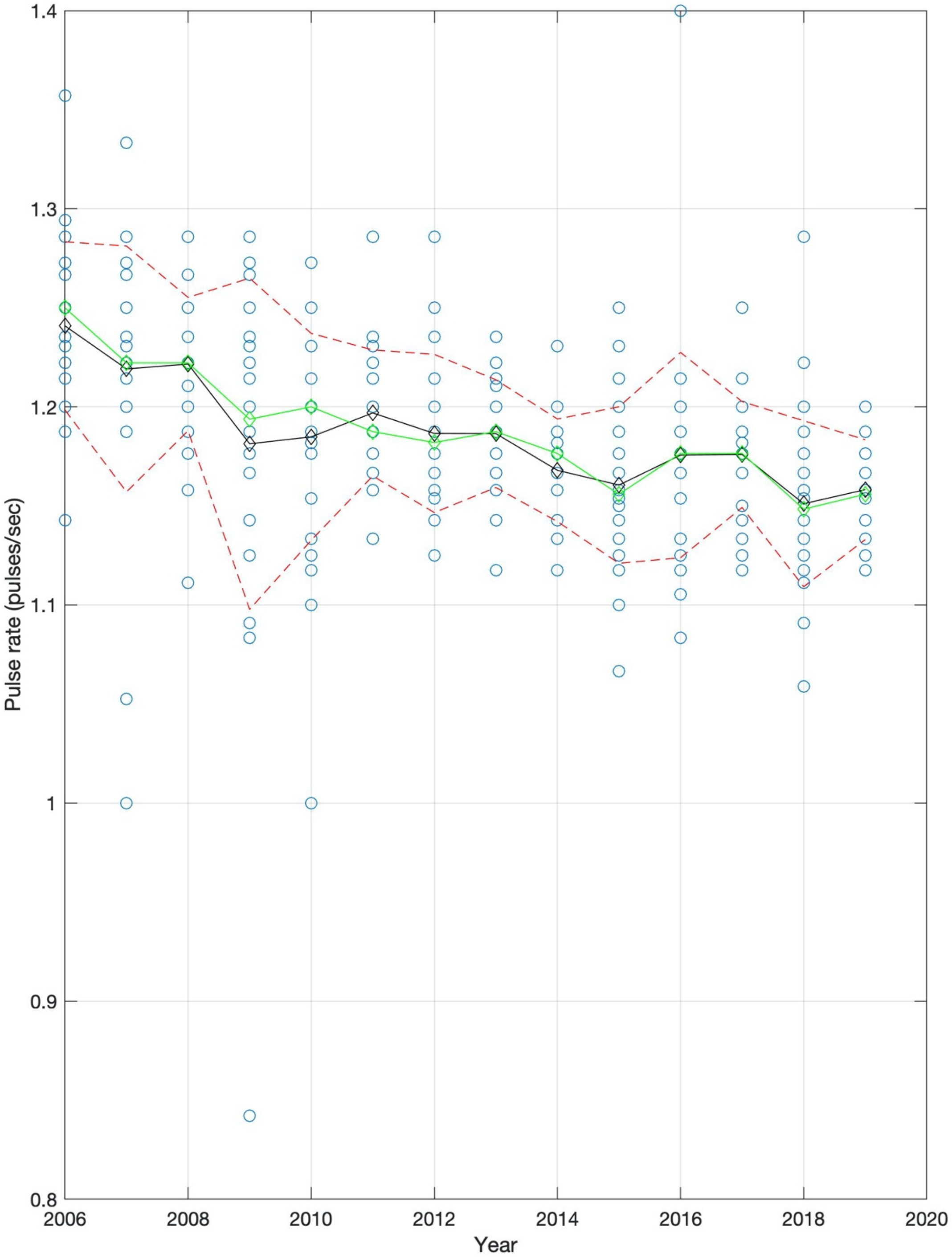

1.4
1.3
1.2
1.1
1
0.9
0.8
Pulse rate (pulses/sec)
2006
2008
2010
2012
2014
2016
2018
2020
Year
